# Association of Tumor Necrosis Factor Inhibitors with the Risk of Nontuberculous Mycobacterial Infection in Patients with Rheumatoid Arthritis: A Nationwide Cohort Study

**DOI:** 10.3390/jcm12226998

**Published:** 2023-11-09

**Authors:** Hyun Jin Park, Boyoon Choi, Yun-Kyoung Song, Yoon-Jeong Oh, Eun Bong Lee, In-Wha Kim, Jung Mi Oh

**Affiliations:** 1 College of Pharmacy and Research Institute of Pharmaceutical Sciences, Seoul National University, Seoul 08826, Republic of Korea; hjpark059@snu.ac.kr (H.J.P.); boyoon1@cha.ac.kr (B.C.); yksong@cu.ac.kr (Y.-K.S.); 2 Department of Pharmacy, College of Pharmacy and Institute of Pharmaceutical Sciences, CHA University, Pocheon-si 11160, Gyeonggi, Republic of Korea; 3 College of Pharmacy, Daegu Catholic University, Gyeongsan-si 38430, Gyeongbuk, Republic of Korea; 4 Division of Rheumatology, Department of Internal Medicine, Seoul National University College of Medicine, Seoul 03080, Republic of Korea; yjgark640@naver.com (Y.-J.O.); leb7616@snu.ac.kr (E.B.L.)

**Keywords:** tumor necrosis factor inhibitors, nontuberculous mycobacteria, infection, rheumatoid arthritis, cohort study

## Abstract

Tumor necrosis factor inhibitors (TNFi) are proposed as a risk factor for nontuberculous mycobacteria (NTM) infection. Limited research investigates NTM infection risk in rheumatoid arthritis (RA) patients treated with TNFi compared to conventional synthetic disease-modifying antirheumatic drugs (csDMARDs), considering other concurrent or prior non-TNFi antirheumatic drugs. We aimed to evaluate the NTM infection risk associated with TNFi using a real-world database. Patients with RA treated with TNFi or csDMARDs between 2005 and 2016 were identified utilizing the Korean National Health Insurance Service database. To minimize potential bias, we aligned the initiation year of csDMARDs for both TNFi and csDMARD users and tracked them from their respective treatment start dates. The association of TNFi with NTM infection risk was estimated in a one-to-one matched cohort using a multivariable conditional Cox regression analysis. In the matched cohort (*n* = 4556), the incidence rates of NTM infection were 2.47 and 3.66 per 1000 person-year in TNFi and csDMARD users. Compared to csDMARDs, TNFi did not increase the risk of NTM infection (adjusted hazard ratio (aHR) 0.517 (95% confidence interval, 0.205–1.301)). The TNFi use in RA patients was not associated with an increased risk of NTM infection compared to csDMARDs. Nevertheless, monitoring during TNFi treatment is crucial.

## 1. Introduction

Rheumatoid arthritis (RA) is a chronic inflammatory autoimmune disease characterized by joint inflammation, joint damage, bone erosion, and long-term extra-articular organ complications such as cardiovascular and respiratory diseases [1,2]. The advancement of biologic DMARDs (bDMARDs), such as tumor necrosis factor inhibitors (TNFi) and targeted synthetic DMARDs (tsDMARDs), has notably improved the clinical outcomes in treatment-refractory RA patients [3,4,5]. Among these bDMARDs and tsDMARDs, TNFi have predominantly emerged as the first-line treatment for managing csDMARD-refractory RA patients [6,7]. However, there are concerns regarding the safety of TNFi due to heightened susceptibility to infection.

Over the past two decades, there has been a significant increase in the global burden of non-tuberculous mycobacterial (NTM) infection, leading to a rising prevalence of the disease, notably impacting elderly individuals [8,9,10,11]. The concern regarding the risk of NTM infection in patients with RA arises from the fact that more than half of the RA population is aged 55 or older [12,13]. Several studies have reported a higher risk of NTM infection in patients with RA when compared to those without RA, with reported adjusted hazard ratios (aHR) of 4.17 (95% confidence interval (CI) 2.61–6.65) [14], aHR 2.07 (95% CI 1.84–2.32) [15], and aHR 6.24 (95% 4.24–9.17) [16]. Moreover, NTM infection primarily affects individuals with pre-existing lung disease and a history of previous tuberculosis (TB) infection [17,18]. TNFi treatment has been identified as a significant risk factor for TB infection (odds ratio (OR) 2.29 (95% CI 1.09–4.78)) [19]. Given the increased risk of NTM infection following TB infection and the elevated TB infection risk associated with TNFi use, it is essential to assess the risk of NTM infection after TNFi use in patients with RA, particularly in areas where TB is prevalent. Additionally, studies have investigated an association between the risk of NTM infection and TNFi use in patients with RA [20,21,22]. However, the studies examining the association have primarily focused on analyzing the risk of NTM infection with TNFi use without comprehensively considering the patients’ treatment history for RA or the concurrent use of other immunosuppressive anti-rheumatic drugs such as csDMARDs, corticosteroids, and NSAIDs, which could potentially have a significant influence on the infection risk [21,22,23].

The exact mechanism underlying the association between NTM infection and TNFi use is still not fully understood. It is hypothesized that the inhibition of the TNF-alpha cytokine interferes with its role in the immune system, which involves the activation of macrophages responsible for killing invading pathogens and the formation of granulomas responsible for controlling the spread of mycobacteria [24,25]. Consequently, the use of TNFi may potentially increase susceptibility to NTM infections. While various studies have reported an association between the risk of NTM infection and TNFi use, there is a limited number of studies that have specifically examined the risk of NTM infection among patients with RA treated with TNFi compared to csDMARDs in a real-world setting. Therefore, the objective of this study was to comprehensively evaluate the risk of NTM infection following TNFi treatment as compared to csDMARD treatment using a real-world nationwide database.

## 2. Materials and Methods

### 2.1. Data Sources

The retrospective, nationwide population-based cohort study was conducted by retrieving data from all patients diagnosed with RA from the Korean National Health Insurance Service (NHIS) database. The Korean National Health Information Database (NHID) is a nationwide administrative insurance database covering approximately 97% of the Korean population since the year 2002. This database includes information on sociodemographic variables and healthcare utilization, including records on inpatient and outpatient usage, as well as prescription records for the entire nationwide population [26]. This study adhered to the Strengthening the Reporting of Observational Studies in Epidemiology (STROBE) recommendations [27].

### 2.2. Ethical Approval

This study was approved by the Institutional Review Board of the Seoul National University Hospital (IRB-1710-112-897). The study was conducted in accordance with the Declaration of Helsinki. Patient written informed consent was waived by the institutional review board due to the de-identified nature of the NHIS database.

### 2.3. Study Cohort

Using a validated algorithm established for the identification of patients with RA within the Korean NHID, individuals with seropositive RA categorized based on the International Classification of Diseases, 10th Revision, Clinical Modification (ICD-10-CM) code (M05.*) and those who had received at least one prescription of biologic or csDMARDs for RA treatment between 2005 and 2016 were identified as true RA patients [28]. Among these identified true RA patients, those who (1) were medical aid benefit patients, (2) had a history of RA treatment during screening period, defined as the three-year period before 2005, (3) had a history of NTM infection before the index date, (4) were younger than 19 years old, (5) used non-TNFi biologics, and (6) received treatment for RA for less than 6 months after the index date or had poor medication adherence of TNFi were excluded from the study. Medical aid benefit patients were excluded due to a substantial amount of missing prescription information in the database. The index date for TNFi users was defined as the initial date of their first TNFi prescription, while for csDMARD users, it was defined as the same date as the first TNFi prescription date in their one-to-one matched TNFi users. Non-TNFi biologics included abatacept, rituximab, tocilizumab, and tofacitinib. Patients treated for less than 6 months with anti-rheumatic treatment were excluded to examine the infection risk associated with the long-term use of TNFi. Poor medication adherence was defined as a proportion of days covered (PDC) below 0.8 [29]. Patients with a PDC of below 0.8 were excluded to minimize confounding factors associated with poor medication adherence among patients and to account for the dose-dependent characteristics of infection as a side effect of TNFi [30].

Patients who initiated TNFi between January 2005 and December 2016 and sustained a treatment period of at least 6 months were identified as TNFi users. Patients who started csDMARDs during the same period with at least 6 months of the treatment period and who had never received TNFi were identified as csDMARD users. TNFi included adalimumab, infliximab, golimumab, and etanercept. csDMARDs included methotrexate, hydroxychloroquine, sulfasalazine, leflunomide, auranofin, azathioprine, bucillamine, cyclophosphamide, cyclosporine, penicillamine, minocycline, mizoribine, mycophenolate mofetil, and tacrolimus. For the purpose of establishing a positive control, the incidence of TB infection was estimated, considering the widely acknowledged association between TNFi use and the risk of TB infection.

### 2.4. Main Outcome and Confounding Variables

**Figure 1:**
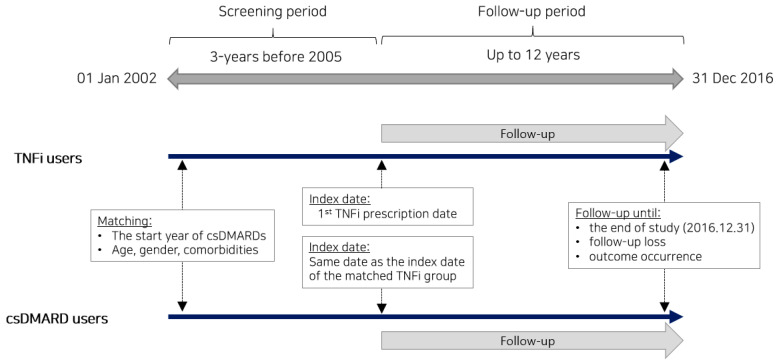
Study design timeline. Jan, January; Dec, December; TNFi, tumor necrosis factor inhibitors; csDMARD, conventional synthetic disease-modifying antirheumatic drugs.

The main outcome of the study was defined as the occurrence of NTM infection, classified by ICD-10 code A31.*. Patients were followed up until the earliest NTM infection event date, loss to follow-up, or 31 December 2016, whichever occurred first (Figure 1).

Potential confounding variables included demographic characteristics (age and gender), comorbid diseases (diabetes mellitus (DM), chronic liver disease (CLD), lung disease (LD), gastroesophageal reflux disease (GERD), cancer (CA), human immunodeficiency virus infection (HIV), solid organ transplantation, history of tuberculosis (TB) infection, and Charlson Comorbidity Index (CCI) score), the duration of csDMARD treatment before the index date, PDC of csDMARDs/nonsteroidal anti-inflammatory drugs (NSAIDs)/oral corticosteroids, and income levels (high, intermediate, and low income). Comorbidities were identified using ICD-10 codes and relevant prescription history during the one-year period before the index date (detailed operational definitions of each comorbid disease are given in Supplementary Material Table S1) [31,32,33,34,35,36,37,38,39]. The duration of csDMARD treatment before the index date served as a surrogate measure for the duration of RA treatment in patients. Income levels were categorized according to the patient’s individual NHI premium quintile and occupation data provided in the NHIS database. 

### 2.5. Statistical Analysis

Baseline characteristics of the study cohort were summarized using descriptive statistics. Continuous variables were presented as mean ± standard deviation, while categorical variables were expressed as frequency (percentage). To achieve a balance between TNFi users and csDMARD users, patients were matched on a one-to-one exact matching on variables including age, gender, and comorbidities (DM, LD, GERD, CA, HIV, transplantation, and history of TB), as well as the initiation year of csDMARDs.

The distribution of baseline covariates between TNFi users and csDMARD users in the matched cohort was evaluated, aiming for a standardized mean difference of <0.1 to ensure a well-balanced distribution. Variables that remained unmatched were included as adjustment variables in all analyses. Multivariable conditional Cox regression was used to calculate the adjusted hazard ratio (aHR) and 95% CIs. Adjusted confounders in the multivariable analyses include CCI, the duration of csDMARD treatment before the index date, PDC of csDMARDs/NSAIDs/oral corticosteroid treatments, as well as income levels.

### 2.6. Subgroup Analyses and Sensitivity Analysis

Stratified subgroup analyses were conducted according to age (<65 and ≥65 years), gender, the duration of csDMARD treatment before the index date (<12 months, 12 ≤ months < 36, and ≥36 months), the specific type of csDMARD treatment, the duration of csDMARD treatment (<18 and ≥18 months), and the duration of follow-up time until the occurrence of NTM infection (<48 and ≥48 months). The subgroup analyses were performed on the matched cohort. The aHR of NTM infection in TNFi users was estimated in each subgroup compared to those in csDMARD users. Further subgroup analyses were conducted according to each type of TNFi treatment and the duration of TNFi treatment (<24 and ≥24 months). The risk of NTM infection in each subgroup of TNFi users were compared to the entire group of csDMARD users. 

To ensure the robustness of the primary findings, we conducted a sensitivity analysis. This sensitivity analysis involved re-evaluating the results by limiting the follow-up period to 3 months after the last TNFi or csDMARD prescription during the study period.

All statistical analyses were performed using SAS software version 9.4 (SAS Institute Inc., Cary, NC, USA). *p*-values < 0.05 were considered statistically significant.

## 3. Results

### 3.1. Baseline Characteristics

**Figure 2:**
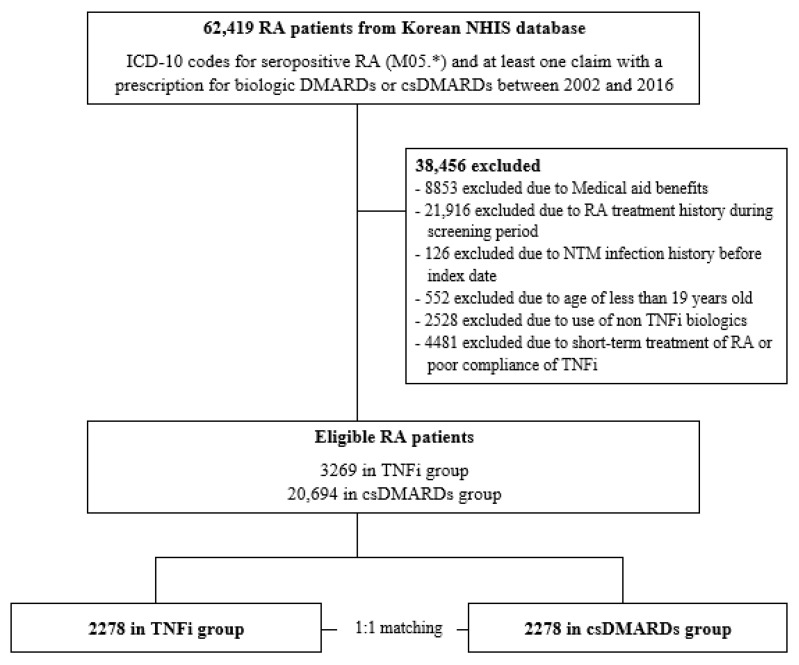
Study flow diagram. RA, rheumatoid arthritis; NHIS, national health insurance service; ICD-10, International Classification of Diseases 10th Revision; DMARDs, disease-modifying antirheumatic drugs; csDMARDs, conventional synthetic disease-modifying antirheumatic drugs; NTM, nontuberculous mycobacteria; TNFi, tumor necrosis factor inhibitors.

A total of 62,419 patients in the NHIS database between 1 January 2005 and 31 December 2016 were identified as seropositive true RA patients. After applying the exclusion criteria, 3269 patients in the TNFi users and 20,694 patients in the csDMARD users were considered eligible seropositive RA patients for the study. Subsequently, after 1:1 matching, the matched cohort included in our study analysis comprised 4556 patients, with 2278 patients in the TNFi users and 2278 patients in the csDMARD users (Figure 2). 

After the 1:1 matching process, the baseline characteristics between TNFi users and csDMARD users were well balanced, with a standardized mean difference below 0.1 for all matched covariates. Baseline characteristics are shown in Table 1 (unmatched cohort table in Supplementary Material Table S2). Among the patients in the matched cohort, approximately 80% were women. The mean age of patients in our study cohort was 51.2 ± 13.0 years. Concerning RA-related baseline characteristics, the mean duration of csDMARD treatment prior to the index date was 35.4 ± 29.9 months, and the mean number of each individual csDMARD treatment was 3.6 ± 1.34 for TNFi users and 3.3 ± 1.37 for csDMARD users, which was similar between the two groups. Among TNFi users, the mean number of each individual TNFi treatment used was 1.2 ± 0.44, and the mean duration of TNFi treatment was 37.7 ± 24.99 months.

### 3.2. The Risk of NTM Infection in TNFi Users Compared to csDMARD Users

In the matched cohort, the incidence rate (IR) of NTM infection was 2.47 events per 1000 person years among TNFi users, while it was 3.66 per 1000 person years in csDMARD users (Table 2) (unmatched cohort table in Supplementary Material Table S3). The aHR for NTM infection in TNFi users was 0.517 (95% CI 0.205–1.301) compared with csDMARD users (Table 3) (unmatched cohort table in Supplementary Material Table S4). These findings indicate that there was no statistically significant association between the use of TNFi and the incidence of NTM infection. Regarding TB infection, which served as a positive control outcome, it was higher among the TNFi users compared to csDMARD users (aHR 7.39, 95% CI: 1.19–45.92; *p* = 0.031) within the initial six months of TNFi use in the matched cohort, highlighting the validity of our analysis. 

### 3.3. Subgroup Analyses and Sensitivity Analysis

Subgroup analyses conducted did not show a significant association between the use of TNFi and the risk of NTM infection across diverse subgroups stratified by patient age groups, gender, the duration of csDMARD treatment before the index date, each TNFi use, duration of TNFi use, each csDMARD use, duration of csDMARD use, and NTM infection time to event (Table 4). Furthermore, a sensitivity analysis limiting the patient follow-up period to three months after the last TNFi or csDMARD prescription during the study period did not demonstrate a significant association between TNFi use and the risk of NTM infection: aHR 0.584 (95% CI 0.310–1.102) (Table 5). These subgroup analyses and sensitivity analysis results reinforced the robustness of our primary findings. 

## 4. Discussion

This study is of great significance in research as it conducts a real-world retrospective cohort analysis to evaluate the risk of NTM infection in RA patients treated with TNFi versus csDMARDs, considering the impact of prior or concurrent use of non-TNFi antirheumatic drugs, using comprehensive nationwide administrative data. This longitudinal study demonstrates that there was no statistically significant difference in the risk of NTM infection in TNFi-treated patients compared to csDMARD-treated patients (aHR 0.517 (95% CI 0.205–1.301)). 

Numerous studies have suggested an increased risk of NTM infection among individuals with RA using TNFi [20,22,23,40]. However, only a few studies have reported no association between TNFi and NTM infection compared to non-users [13,41]. Liao et al. [13] found no significant difference in the risk of NTM infection between RA patients treated with TNFi and those who did not use TNFi (adjusted odds ratio (aOR) = 2.03, 95% CI 0.85–4.86). Takei et al. [41] reported no significant association between NTM infection and bDMARDs, including TNFi (*p* = 0.25). However, Takei et al. [41] implied that RA disease activity itself (*p* < 0.01) and the use of glucocorticoid (aHR 2.5 (95% CI 1.5–4.3) were potential risk factors for NTM infection, which were adjusted in our study analysis. In addition, previous studies that have reported an increased risk of NTM infection primarily relied on case–control studies to establish associations without directly examining NTM infection incidence and prevalence [13,22,42]. For instance, Brode et al. [22] reported TNFi treatment as a potential risk factor for an elevated risk of NTM infection (aOR 2.19 (95% CI 1.10–4.37)) among RA patients who had at least one prescription for csDMARDs, biological agents, or NSAIDs, as determined by Canadian administrative data. Park et al. [23] was the only retrospective cohort study evaluating the association between the use of TNFi and the risk of NTM infection. Park et al. [23] reported an increased risk of NTM infection in TNFi-treated RA patients as compared to non-TNFi-treated RA patients (aHR 1.751 (95% CI 1.105–2.774)) within the Korean population, based on administrative data. 

The incidence rate of NTM infection observed in our study among TNFi-treated RA patients (IR 2.47 per 1000 person year) was comparable to findings from other studies conducted in Korea: Lee et al. [21] reported an IR of 230.7 per 100,000 person years in a single center, while Park et al. [23] reported an IR of 328.1 per 100,000 person years in the Korean RA population. In our study, the follow-up duration for TNFi users averaged 3.9 ± 2.2 years, and for csDMARD users, it averaged 3.8 ± 2.2 years. This duration proved sufficient to evaluate NTM infection incidence, aligning with previous research indicating that NTM infection typically occurs within the first three years after TNFi initiation [23,43]. However, the inconsistency between our research findings on NTM infection risk and those of previous studies may be attributed to differences in the study design. Notably, the distinct definition of the comparator group (TNFi unexposed group) and the index date would definitely generate these inconsistencies. Park et al. [23] defined the comparator group as all RA patients except those using TNFi and used the date of the first RA diagnosis as the index date for TNFi untreated RA patients. It is conceivable that the TNFi unexposed group might have included a higher proportion of immunocompetent patients compared to our study. Hence, the risk of NTM infection, known to be elevated in immunocompromised patients [44], might have been lower in their TNFi unexposed group. Additionally, the difference in the index dates for the TNFi unexposed group (csDMARD users) in our study might have exerted a significant influence on the risk of NTM infection. Brode et al. [22] comprised a distinct study population from our study, as their research focused on elderly RA patients aged 67 years and older who had been prescribed at least one prescription for csDMARDs, biological agents, or NSAIDs. This included a portion of patients who were treated solely with NSAIDs as well. These differences in the definition of the TNFi unexposed group, along with population age difference, might have potentially contributed to an increased risk of NTM infection in the TNFi user group.

Moreover, it is important to note that previous studies did not account for the potential impact of other prior or concurrent use of immunosuppressive non-TNFi antirheumatic drugs, such as csDMARDs, oral corticosteroids, and non-TNFi biologics, on the risk of NTM infection in their analysis [13,22,23]. The lack of this consideration contributes to variations in the observed NTM infection risk across different studies. The presence and interaction of these immunosuppressive anti-rheumatic drugs can significantly influence the overall susceptibility to NTM infection. The absence of their consideration in previous research introduces confounding variables that impact result interpretation. In contrast, our study comprehensively accounts for the potential influence of these immunosuppressive anti-rheumatic drugs, thereby enhancing the comprehensiveness of the analysis and providing a more robust assessment of the association between TNFi use and NTM infection risk.

By selecting csDMARD users as the comparator and matching the start year of initial csDMARD between TNFi users and csDMARD users while defining the index date as the first prescription date of TNFi for TNFi users and its corresponding matching date for csDMARD users, our study effectively mitigated the risk of immortal time bias. This rigorous approach enabled us to accurately assess the influence of TNFi exposure on the occurrence of NTM infection. By addressing this potential bias, we enhanced the validity and reliability of our findings regarding the relationship between TNFi usage and the risk of NTM infection.

The precise mechanism associated with TNFi and NTM infection in RA patients remains to be fully elucidated. The suggested mechanisms involve immune deficiency resulting from the inhibition of TNF-α, which is essential for the formation of granuloma necessary to control mycobacterial infection [45]. This inhibition could potentially create an environment conducive to mycobacterial proliferation. Additionally, TNFi may disrupt the activation of macrophages and the formation of phagosomes, which are critical for eliminating intracellular pathogens like NTM [46] and increase susceptibility to opportunistic infections caused by neutropenia [47]. All these mechanisms offer potential insights into the complex interplay between TNFi usage and the risk of NTM infection in RA patients. We employed a matching technique to align with previously reported risk factors for NTM infection (TB infection history and lung disease) as well as the initiation year of the first csDMARDs [13]. Additionally, adjustments were made for the PDC in oral corticosteroid treatment and the duration of csDMARD treatment before the index date. This approach distinguished our analysis from prior investigations. Furthermore, the absence of a significant NTM risk in our study could be attributed to physicians possibly prescribing fewer instances of TNFi for patients deemed to possess a heightened risk for NTM infection.

This study has several notable strengths. First, it includes a real-world population-based NHID in the analysis, encompassing all patients with RA in Korea from 2002, the first available year of the NHIS-NHID, to 2016, and including all TNFi claims since its first approval in 2005 in Korea. Patient data in NHID were retrieved from 2002, considering the three-year screening period. This comprehensive approach enabled an assessment that spans the entirety of all RA patients in Korea, effectively minimizing the selection bias. Moreover, this study evaluated the long-term effect of TNFi by including patients who underwent TNFi therapy for a minimum of six months while maintaining good medication adherence, with all patients using TNFi having a PDC greater than or equal to 0.8. In actual real-world clinical settings, TNFi is typically used for the long term in patients with RA, with a median treatment duration of 26 months in Korea [48]. Also, in Korea, the median duration from initiation of TNFi to the development of NTM infection has been reported to be approximately 29.4 months [23]. It is worth noting that while infections commonly occur within 6–12 months of initiation, some studies have reported a continued risk of serious infections even after long-term use of TNFi [49,50,51,52]. Therefore, it is necessary to evaluate the risk of NTM infection during long-term TNFi use, as this represents a research gap that warrants further investigation. Additionally, the exclusion of patients with a PDC below 0.8 was to minimize potential confounding bias related to confounding factors associated with poor medication adherence and to account for the dose-dependent characteristics of infection as a side effect of TNFi. By matching the initiation year of csDMARD use and defining the initial prescription date of TNFi, along with its corresponding matching date as the respective study index dates for TNFi and csDMARD users, our study was able to avoid potential immortal time bias. This study design reflects the reimbursement criteria for TNFi in Korea, which is approved for RA patients who are refractory to csDMARD treatment. This study reflects the strict reimbursement criteria for TNFi treatment in Korea until recently, which resulted in a delayed initiation of TNFi treatment for TNFi users (33.8 ± 29.79 months) [48,53]. Furthermore, as a means of validating our study findings, the risk of TB infection was evaluated as a positive control. This analysis revealed a significant increase in TB infection risk among TNFi users, a well-recognized risk factor for NTM infection. However, it should be noted that further research is warranted to confirm our study’s results, specifically in relation to the absence of an association between TNFi use and NTM infection risk.

This study has several limitations that should be acknowledged. First, due to the inherent nature of the administrative data, patients’ clinical and laboratory data on RA disease activity were not fully accounted for in the analysis, potentially resulting in misclassification. Although efforts to match the initial csDMARDs start the year and adjust for PDC of csDMARDs/NSAIDs/oral corticosteroids to control for potential confounders, unadjusted confounders might have still impacted the overall NTM infection risk. This limitation could be addressed by establishing data linkage between clinical data and claims data in future studies. Secondly, given the relatively low incidence of NTM infection in general, the size of the study sample may not have been robust enough to yield sufficient statistical power for the detection of a significant effect. Nonetheless, it is important to highlight that this study included all available RA patients in Korea, enhancing the generalizability of the findings and making them more representative of the risk in the Korean RA population. This broad inclusion ensures a comprehensive assessment of the association between TNFi and csDMARD use and the risk of NTM infection in this particular population. Additionally, it is worth noting that our study defined the study population as individuals who had used TNFi or csDMARDs for a minimum of 6 months, leading to the exclusion of patients with treatment durations shorter than this threshold. As a result, patients who used TNFi or csDMARDs for less than 6 months were excluded from the study. This decision was made to effectively assess the long-term safety implications of TNFi or csDMARDs. This choice of a 6-month minimum treatment duration was supported by its suitability in gauging the enduring safety effects of these medications. Furthermore, it is worth mentioning that in the context of RA patients in Korea, the reported treatment duration for TNFi was 26 months on average [48]. This finding indicates that a significant proportion of individuals who use TNFi have been included in our study’s scope. 

## 5. Conclusions

In conclusion, the findings of this study suggest that the utilization of TNFi among patients with RA is not significantly associated with an increased risk of NTM infection when compared to csDMARD use, taking into account the prior or concurrent use of other non-TNFi antirheumatic drugs. Nonetheless, it is imperative to exercise vigilance and diligent monitoring of patients while on TNFi treatment. While these results provide valuable insights, further studies are needed to confirm the study results and to suggest treatment strategies for RA patients in actual clinical practice settings.

## Figures and Tables

**Table 1:** Baseline characteristics of the matched cohort.

Variables	Matched Cohort		
	TNFi n = 2278	csDMARDs n = 2278	SMD
Female gender	1815 (79.6)	1815 (79.6)	0
Age (years)	51.2 ± 13.04	51.2 ± 13.04	0
Comorbidities *			
Diabetes	123 (5.3)	123 (5.3)	0
CLD	200 (8.7)	200 (8.7)	0
LD	329 (14.4)	329 (14.4)	0
GERD	500 (21.9)	500 (21.9)	0
ISD	24 (1.0)	24 (1.0)	0
hTB	1 (0.04)	1 (0.04)	0
Number of comorbid diseases			0
0	1320 (57.9)	1320 (57.9)	
1	759 (33.3)	759 (33.3)	
2 or more	199 (8.7)	199 (8.7)	
Charlson comorbidity index score			-
1	702 (30.8)	753 (33.0)	
2	707 (31.0)	641 (28.1)	
3 or more	869 (38.1)	884 (38.8)	
Duration of csDMARD treatment before the index date (months)	35.4 ± 29.92	35.3 ± 29.96	0.003
TNFi treatment **			
Adalimumab	1097 (48.1)	-	
Etanercept	860 (37.7)	-	
Golimumab	279 (12.2)	-	
Infliximab	467 (20.5)	-	
Duration of TNFi treatment (months)	37.7 ± 24.99	-	
PDC of TNFi	0.98 ± 0.043	-	
csDMARD treatment **			
Methotrexate	2130 (93.5)	1756 (77.0)	
Hydroxychloriquine	1831 (80.3)	1914 (84.0)	
Sulfasalazine	1595 (70.0)	1143 (50.1)	
Leflunomide	1263 (55.4)	872 (38.2)	
Number of csDMARDs	3.6 ± 1.34	3.3 ± 1.37	
Duration of csDMARDs (months)	40.3 ± 26.77	35.5 ± 25.98	
PDC of csDMARDs	0.88 ± 0.284	0.80 ± 0.337	
Anti-inflammatory treatment **			
PDC of oral corticosteroid	0.75 ± 0.346	0.66 ± 0.387	
PDC of NSAIDs	0.86 ± 0.251	0.72 ± 0.351	
Type of institution			-
Tertiary hospital	2099 (92.1)	1539 (67.5)	
General hospital	118 (5.1)	274 (12.0)	
Community hospital/clinics/others	61 (2.6)	465 (20.4)	
Income levels ***			-
High	668 (29.3)	564 (24.7)	
Intermediate	906 (39.7)	959 (42.0)	
Low	704 (30.9)	755 (33.1)	

Values are represented as frequency (percent) or mean ± standard deviation; * Comorbidities and the Charlson comorbidity index scores were determined during one-year period prior to index date; ** RA treatments were determined from the study index date to the end of follow-up; *** Income levels were categorized according to patient’s individual NHI premium quintile and occupation data provided in the NHIS database; TNFi, tumor necrosis factor inhibitor; csDMARDs, conventional synthetic disease-modifying anti-rheumatic drugs; SMD, standardized mean difference; CLD, chronic liver disease; LD, lung disease; GERD, gastroesophageal reflux disease; ISD, immunosuppressive disease; hTB, history of tuberculosis infection; PDC, proportion of days covered; NSAIDs, non-steroidal anti-inflammatory drugs.

**Table 2:** Incidence rates of nontuberculous mycobacteria (NTM) infection on matched cohort.

	Matched Cohort	
	TNFi	csDMARDs
NTM infection event number	22	32
IR (1000 person years)	2.47	3.66

TNFi, tumor necrosis factor inhibitors; csDMARDs, conventional synthetic disease-modifying anti-rheumatic drugs; NTM, nontuberculous mycobacteria; IR, incidence rate.

**Table 3:** Adjusted hazard ratio for nontuberculous mycobacteria (NTM) infection on matched cohort.

	Matched Cohort			
Variables	aHR	95% CI		*p*-Value
TNFi treatment	0.517	0.205	1.301	0.161
Charlson comorbidity index score	0.703	0.323	1.528	0.373
Duration of csDMARD treatment before the index date (months)	0.960	0.803	1.147	0.650
RA treatments				
Number of csDMARDs	1.775	0.750	4.202	0.191
PDC of csDMARDs	0.015	0.001	0.388	0.011
PDC of oral corticosteroid	0.754	0.093	6.127	0.792
PDC of NSAIDs	5.646	0.633	50.364	0.121
Methotrexate user	0.427	0.059	3.112	0.401
Hydroxychloroquine user	1.075	0.130	8.880	0.946
Sulfasalazine user	0.268	0.070	1.023	0.054
Leflunomide user	0.864	0.198	3.772	0.845
Income levels				
High vs. low	1.309	0.332	5.158	0.701
Intermediate vs. low	0.347	0.097	1.238	0.103

aHR, adjusted hazard ratio; CI, confidence interval; TNFi, tumor necrosis factor inhibitors; csDMARDs, conventional synthetic disease-modifying anti-rheumatic drugs; RA, rheumatoid arthritis; PDC, proportion of days covered; NSAIDs, non-steroidal anti-inflammatory drugs.

**Table 4:** Subgroup analyses for adjusted hazard ratio for nontuberculous mycobacteria (NTM) infection.

Variables	aHR	95% CI		*p*-Value
Age group *				
Less than 65	0.665	0.330	1.337	0.252
65 or more	1.176	0.386	3.584	0.775
Gender *				
Female	0.648	0.336	1.251	0.196
Male	1.591	0.317	7.982	0.572
Duration of csDMARD treatment before the index date (months) *				
<12 months	1.069	0.366	3.121	0.903
12 ≤ months < 36	1.415	0.491	4.084	0.520
≥36 months	0.349	0.116	1.047	0.060
TNF inhibitor **				
Adalimumab	0.596	0.291	1.220	0.156
Etanercept	1.351	0.417	4.377	0.616
Infliximab	1.224	0.262	5.721	0.797
Duration of TNF inhibitor use **				
<24 months	1.051	0.320	3.454	0.935
≥24 months	0.635	0.317	1.271	0.199
csDMARDs *				
Methotrexate	0.773	0.412	1.450	0.422
Hydroxychloroquine	0.655	0.343	1.251	0.199
Sulfasalazine	0.683	0.319	1.462	0.326
Leflunomide	0.893	0.389	2.052	0.790
Duration of csDMARD use *				
<18 months	0.802	0.359	1.795	0.591
≥18 months	0.765	0.311	1.877	0.557
Time to NTM infection incidence *				
<48 months	1.039	0.519	2.084	0.913
≥48 months	0.381	0.110	1.322	0.128

* Comparator defined as the subgroups in the csDMARD users; ** comparator defined as the entire csDMARD users; aHR, adjusted hazard ratio; CI, confidence interval; csDMARDs, conventional synthetic disease-modifying anti-rheumatic drugs; TNF, tumor necrosis factor; NTM, nontuberculous mycobacteria.

**Table 5:** Sensitivity analysis for adjusted hazard ratio for nontuberculous mycobacteria (NTM) infection according to NTM infection monitoring time.

Variables	aHR	95% CI		*p*-Value
NTM infection monitoring time				
3 months	0.584	0.310	1.102	0.097

aHR, adjusted hazard ratio; CI, confidence interval; NTM, nontuberculous mycobacteria.

## Data Availability

The computing code required to replicate the results is provided on request. We cannot provide data from the National Health Insurance Database due to data user agreement, but the data could be requested from the National Health Insurance Service.

## Supplementary Materials

The following supporting information can be downloaded at https://www.mdpi.com/article/10.3390/jcm12226998/s1. Table S1: Detailed operational definitions of each comorbid disease. Table S2. Baseline characteristics of unmatched cohort. Table S3. Incidence rates of nontuberculous mycobacteria (NTM) infection on unmatched cohort. Table S4. Adjusted hazard ratio for nontuberculous mycobacteria (NTM) infection on unmatched cohort.
