# Supplementary material for: Association of Tumor Necrosis Factor Inhibitors with the Risk of Nontuberculous Mycobacterial Infection in Patients with Rheumatoid Arthritis: A Nationwide Cohort Study
Source: J Clin Med. 2023 Nov 9;12(22):6998. doi: 10.3390/jcm12226998 (PMC10671978; doi:10.3390/jcm12226998)
Supplement: Supplementary file 1 [file jcm-12-06998-s001.zip › jcm-2692579-supplementary.pdf]

Table S1. Detailed operational definitions of each comorbid disease.

Comorbid disease	Operational definition
Diabetes mellitus	Diagnostic code (diabetic mellitus E10.*-E14.*) and antidiabetic medication prescription code
Chronic liver disease	Diagnostic code (acute hepatitis B15.*, B16.*, viral hepatitis B17.*-B19.*, alcoholic liver disease K70.*, chronic hepatitis K73.*, fibrosis and cirrhosis of liver K74.*)
Lung disease	Diagnostic code (emphysema J43.*, chronic obstructive pulmonary disease J44.*, except MacLeod's syndrome J430) and prescription code for methylxanthine or inhaler in patients who are 40 years and older<br>Diagnostic code (pulmonary aspergillosis B44.*, cystic fibrosis E84.*, pneumonia J12.0*-J18.*, asthma J45.*-J46.*, bronchiectasis J47.*, interstitial pulmonary disease J84.*)
Gastroesophageal reflux disease	Diagnostic code (gastro-esophageal reflux disease K21.*) and proton-pump inhibitor prescription for more than 14 days
Cancer	Diagnostic code (malignant neoplasm C00.*-C97.*) and cancer treatment history (chemotherapy, radiotherapy, surgery)
Human immunodeficiency virus (HIV) infection	Diagnostic code (human immunodeficiency virus disease B20.*-B24.*)
Solid organ transplantation	Diagnostic code (solid organ transplantation Z94.*)
Tuberculosis history	More than two diagnostic code (tuberculosis A15.*-A19.*, U84.*) and more than three prescription code for tuberculosis treatment medication

Table S2. Baseline characteristics of unmatched cohort.

Variables	Unmatched cohort		
	**TNFi n=3269**	**csDMARD n=20,694**	**SMD**
Female gender	2287 (69.9)	15,088 (72.9)	0.065
Age (years)	50.4 ± 14.00	57.2 ± 14.20	0.486
Comorbidities*			
Diabetes	322 (9.8)	2877 (13.9)	-0.125
CLD	565 (17.2)	3451 (16.6)	0.016
LD	637 (19.4)	5355 (25.8)	-0.153
GERD	949 (29.0)	2745 (13.2)	0.393
ISD	0 (0)	907 (4.3)	-0.302
hTB	61 (1.8)	331 (1.5)	0.020
Number of comorbid diseases			0.018
0	1489 (45.5)	9869 (47.6)	
1	1187 (36.3)	7273 (35.1)	
2 or more	593 (18.1)	3552 (17.1)	
Charlson comorbidity index score			-
1	855 (26.1)	4511 (21.7)	
2	947 (28.9)	5069 (24.4)	
3 or more	1467 (44.8)	11,114 (53.7)	
Duration of csDMARDs treatment before the index date (months)	33.8 ± 29.79	-	-
TNFi treatment**			
Adalimumab	1567 (47.9)	-	
Etanercept	1259 (38.5)	-	
Golimumab	399 (12.2)	-	
Infliximab	669 (20.4)	-	
Duration of TNFi treatment (months)	37.6 ± 25.15	-	
PDC of TNFi	0.98 ± 0.043	-	
csDMARDs treatment**			
Methotrexate	2968 (90.7)	15,705 (75.8)	
Hydroxychloriquine	2526 (77.2)	17,191 (83.0)	
Sulfasalazine	2344 (71.7)	10,103 (48.8)	
Leflunomide	1740 (53.2)	7364 (35.5)	
Number of csDMARDs	3.6 ± 1.41	3.1 ± 1.35	
Duration of csDMARDs (months)	38.5 ± 26.84	50.4 ± 35.63	
PDC of csDMARDs	0.85 ± 0.311	0.71 ± 0.309	
Anti-inflammatory treatment**			
PDC of oral corticosteroid	0.73 ± 0.359	0.56 ± 0.354	
PDC of NSAIDs	0.85 ± 0.264	0.62 ± 0.341	
Type of institution			-
Tertiary hospital	3004 (91.8)	14,147 (68.3)	
General hospital	176 (5.3)	2487 (12.0)	
Community hospital/ clinics/others	89 (2.7)	4060 (19.6)	
Income levels***			-
High	921 (28.1)	6144 (29.6)	
Intermediate	1319 (40.3)	8411 (40.6)	
Low	1029 (31.4)	6139 (29.6)	

Values are represented as frequency (percent) or mean ± standard deviation; * Comorbidities and the Charlson comorbidity index scores were determined during one-year period prior to index date; ** RA treatments were

determined from the study index date to the end of follow-up; *** Income levels were categorized according to patient's individual NHI premium quintile and occupation data provided in the NHIS database; TNFi, tumor necrosis factor inhibitors; csDMARD, conventional synthetic disease-modifying anti-rheumatic drugs; SMD, standardized mean difference; CLD, chronic liver disease; LD, lung disease; GERD, gastroesophageal reflux disease; ISD, immunosuppressive disease; hTB, history of tuberculosis infection; PDC, proportion of days covered; NSAIDs, non-steroidal anti-inflammatory drugs.

Table S3. Incidence rates of nontuberculous mycobacteria (NTM) infection on unmatched cohort.

	Unmatched cohort	
	**TNFi**	**csDMARD**
NTM infection event number	35	438
IR (1000 person years)	2.77	3.39

TNFi, tumor necrosis factor inhibitors; csDMARD, conventional synthetic disease-modifying anti-rheumatic drugs; NTM, nontuberculous mycobacteria; IR, incidence rate.

Table S4. Adjusted hazard ratio for nontuberculous mycobacteria (NTM) infection on unmatched cohort.

Variables	Unmatched cohort			
	**aHR**	**95% CI**		**p-value**
TNFi treatment	0.893	0.525	1.518	0.675
Age (years)	1.223	1.133	1.320	<0.001
Male gender	1.266	1.039	1.543	0.020
Comorbidities*				
Diabetes	0.638	0.474	0.860	0.003
CLD	1.094	0.863	1.388	0.457
LD	2.221	1.825	2.702	<0.001
GERD	1.159	0.888	1.514	0.277
ISD	1.395	0.813	2.394	0.226
hTB	3.362	2.269	4.983	<0.001
Charlson comorbidity index score	1.122	0.975	1.292	0.108
Duration from the initial csDMARDs start date to the index date (months)	0.999	0.986	1.012	0.892
RA treatments				
Number of csDMARDs	1.139	1.053	1.234	0.001
PDC of csDMARDs	1.038	0.679	1.589	0.862
PDC of oral corticosteroid	2.197	1.509	3.199	<0.001
PDC of NSAIDs	0.953	0.694	1.309	0.767
Income levels				
High vs. low	1.423	1.116	1.813	0.004
Intermediate vs. low	1.181	0.933	1.495	0.167

aHR, adjusted hazard ratio; CI, confidence interval; TNFi, tumor necrosis factor inhibitors; CLD, chronic liver disease; LD, lung disease; GERD, gastroesophageal reflux disease; ISD, immunosuppressive disease; hTB, history of tuberculosis infection; csDMARDs, conventional synthetic disease-modifying anti-rheumatic drugs; RA, rheumatoid arthritis; PDC, proportion of days covered; NSAIDs, non-steroidal anti-inflammatory drugs.
